# Cincho-Quinine

**Published:** 1874-11

**Authors:** H. B. Briggs

**Affiliations:** Westerly, R. I.


### Cincho-Quinine.

By H. B. Briggs, M. D., of Westerly, R. I.

The use of cincho-quinine as a substitute for the sulphate of quinia was first brought to my notice in August, 1872. Since that time I have used it with seemingly good results, and as its use at the present time is a subject of comment among numerous physicians, I herewith add my own experience with the comparatively new drug.

As a remedy which possesses the power of arresting morbid periodical movements, its use, in my hands, has been decidedly flattering. Although my experience with it in intermittent fever has, of necessity, (on account of locality), been very limited, yet I have used it in several cases, and in every one the results have been good, and in no case has the patient complained of any disagreeable sensation in the head from its use. In several cases of facial neuralgia, the pain occurring in paroxysms, it has been given with decided good results. In one case, the patient a female, the sulphate of quinia was given first, but had to be abandoned on account of the great amount of nausea and vomiting produced. She was put upon the cincho, same dose, and the above unpleasant symptoms vanished. The following case is given as illustrative of its use in simple remittent fever:—

Was called, May 7th, at 10 A. M., to see a patient, C., æt. 34. Found him in bed, suffering fron a severe chill; pulse 110; intense cephalalgia, thirst, bowels constipated, slight nausea and epigastric uneasiness, pain in back, respiration hurried, although free, tongue furred, no appetite, face flushed, urinary seceetion scanty and quite high-colored. The patient's history was thus: Had not felt as well as usual for the past week or ten days. On the day previous, (May 6th), had experienced a chill, which was followed by increased heat of body, and moisture, also by a severe and throbbing headache.

Was ordered comp. cath. pil. iij., to be taken at once. Also the following mixture:—

℞. Tr. aconit. rad., mlx.

Potas. nitrat, ʒiij.

Aquæ puræ, fl.℥ij. M.

Sig. ʒj. every four hours.

Also: ℞. Cincho-quinine, ℈j. Ft. chart, x.

Sig. One powder every four hours, to alternate with above mixture.

May 8th, 3 o'clock, P. M. The patient feels about the same as yesterday. Had a chill about 10 o'clock A. M., although not as severe as yesterday's. Pulse 98; headache continuous; bowels have moved once, stool very dark; nausea continues; has vomited once or twice. Ordered seidlitz powder; yesterdays treatment to be continued. Also, powder, cincho-quinine, gr. x, to be taken at 6 o'clock, A. M., next day.

May 9th, 5 o'clock, P. M. Patient feels much better; has had no chill; pulse 88; bowels moved several times; little or no pain in head or back. Ordered Powders, cincho-quinine, grs. ij, every four hours.

May 10th, 5 o'clock, P. M. Patient feels very much better. Pulse 70; no chill; bowels open; no nausea; tongue clean; appetite not very good. Ordered the following:

℞. Cincho-quinine, ʒss.

Tr. gent. co.,

Syrup simplicis, aa fl.℥ij. M.

Sig. ʒj. in water, three times a day, before eating.

Visits discontinued.

The above case is one of quite a number treated with the cincho during the past two years, with the same good results. As a tonic, could I have but one preparation, I should prefer the cincho in place of the sulphate. In more than one case have we seen the sulphate disappoint, when a good result followed upon the use of the cincho; although the alkaloids may possess the anti-intermittant power of the bark, it has certainly not been proven that they exert all the peculiar influence of that medicine as a tonic; in fact, clinical experience has proven the opposite. Now, if any one of the salts of cinchona does not possess that property, and they all combined do, it is plain that a drug containing them all, or nearly all, must have a wider scope for action than those containing but a single salt. Such a drug we believe the cincho to be, and shall be disappointed if it does not so prove itself in the hands of our professional brethren.

It is with pleasure that I recall its use in typhoid fever; notes of thirty two cases of the above fever that have come under my observation during the past two years, in which use had been made of the cincho, but two proved fatal. The treatment was based upon the supposition that typhoid fever was due to a blood poison, which poison exhausts itself in a few weeks at the farthest, if life can be prolonged until that time, and that we have not, or at least do not know at the present time, of a specific. The treatment consisted in every case, of nourishment, alcoholic stimulus, and tonics. Of the thirty-two cases, the cincho was the only tonic used, with but one exception. In typhoid, one of the greatest dangers is from the severity of the fever. In all fevers of long duration the protracted increased transformation of tissue, on which the feverish overheating depends, induces consumption of the body of the patient. For moderating this fever, the cincho has appeared to be a most efficient remedy. Do not understand me to say that it possesses any abortive properties; the only effect it has upon the disease is to moderete the fever. I usually prescribe one or two grains at a dose, in solution with dilute sulphuric or nitro-hydrochloric acid.

In children, who often show a marked repugnance for the sulphate, the cincho is taken readily, and is seldom if ever followed by any marked gastric disturbance.—*Phila. Med. and Surg. Reporter.*

